# Silkworm Protein-Derived Nitrogen-Doped Carbon-Coated Li[Ni_0.8_Co_0.15_Al_0.05_]O_2_ for Lithium-Ion Batteries

**DOI:** 10.3390/nano12071166

**Published:** 2022-03-31

**Authors:** Gyu Sang Sim, Nitheesha Shaji, P. Santhoshkumar, Jae Woo Park, Chang Won Ho, Murugan Nanthagopal, Hong Ki Kim, Chang Woo Lee

**Affiliations:** 1 Department of Chemical Engineering (Integrated Engineering), College of Engineering, Kyung Hee University, 1732 Deogyeong-daero, Giheung, Yongin 17104, Gyeonggi, Korea; simgyusang0215@khu.ac.kr (G.S.S.); nitheesha@khu.ac.kr (N.S.); jwpark82@khu.ac.kr (J.W.P.); ghckddnjs@khu.ac.kr (C.W.H.); nanthamurugan@khu.ac.kr (M.N.); hkkim95@khu.ac.kr (H.K.K.); 2 Center for the SMART Energy Platform, College of Engineering, Kyung Hee University, 1732 Deogyeong-daero, Giheung, Yongin 17104, Gyeonggi, Korea; santhoshkumar.palanisamy@gmail.com

**Keywords:** Li[Ni_0.8_Co_0.15_Al_0.05_]O_2_, sericin, N-doped carbon, lithium-ion batteries, surface wrapping

## Abstract

Li[Ni_0.8_Co_0.15_Al_0.05_]O_2_ (NCA) is a cathode material for lithium-ion batteries and has high power density and capacity. However, this material has disadvantages such as structural instability and short lifespan. To address these issues, herein, we explore the impact of N-doped carbon wrapping on NCA. Sericin, an easily obtained carbon- and nitrogen-rich component of silk cocoons, is utilized as the precursor material. The electrochemical performance evaluation of N-doped carbon-coated NCA shows that the capacity retention of 0.3 NC@NCA at 1C current density is 69.83% after 200 cycles, which is about 19% higher than the 50.65% capacity retention of bare NCA. The results reveal that the sericin-resultant N-doped carbon surface wrapping improves the cycling stability of NC@NCA.

## 1. Introduction

With the expansion of various devices, there has been a growing demand for various energy storage devices that may be utilized as energy sources [1,2]. Lithium-ion batteries (LIBs) have become the most frequently utilized secondary batteries among energy storage systems due to their attractive properties such as good energy density, operating potential, and extended cycle life [3,4]. The cathode is a crucial part of a battery that influences energy density, power density, longevity, and potential of the battery; it also accounts for 40% of the overall battery price [5,6]. The cathode materials of LIBs including LiCoO_2_ (LCO), LiMn_2_O_4_ (LMO), and LiFePO_4_ (LFP) are those with layered, spinel, and olivine structures, respectively [7,8,9]. Recently, layer structured, high nickel content cathodes, such as Li[Ni_0.8_Co_0.15_Al_0.05_]O_2_ (NCA) and Li[Ni_0.8_Co_0.1_Mn_0.1_]O_2_ (NCM811), have obtained widespread consideration because of their high capacity, relatively inexpensive price, and eco-friendly nature [10,11]. Among them, NCA has been popularized in the battery industry for its low price, high capacity, and power density [11,12]. However, NCA has many problems including poor lifespan, and low thermal and structural stability. Additionally, the degradation of NCA caused by side reactions with the electrolyte is also a major drawback [13,14].

There have been various attempts to address the drawbacks of NCA. Among these attempts, the active material surface wrapping can form a barrier to effectively protect the active material from an unstable structure by suppressing the surface degradation of the material and side reactions with the electrolyte; this improves the structural stability of the material [15,16,17,18]. Depending on the coating material used, the surface wrapping approach can also improve the overall conductivity of the electrode material [19,20].

Carbonaceous materials with wide availability, low cost, high conductivity, and diverse structure have gained significant research interest. Among them, N-doped carbon is an effective material for surface modification of various active materials because it efficiently suppresses surface degradation, prevents by-products from unnecessary side reactions with electrolytes, and improves the electronic conductivity of the host material [21,22,23]. Recently, Feng et al. reported on polyacrylonitrile (PAN)-induced conductive carbon coating on NCA. The PAN-induced carbon coated NCA, with a 5 nm thick coating layer, offered faster electron movement and hence provided an enhanced cycling performance [24]. Ion-conductive polymer Nafion-coating on NCA was investigated by Yigitalp et al. to obtain superior electrochemical performance. They concluded that Nafion-coating could suppress the passive layer formation and provide improved cycling stability [25]. Park et al. explored the conformal graphene coating on NCA by a scalable Pickering emulsion method. The conformal graphene coating reduces the surface degradation of NCA and hence delivered an excellent life cycle [26]. In this study, we utilized sericin to coat N-doped carbon on active materials; it is a biomaterial that is a component of silk cocoons. Sericin, a gummy component that surrounds fibroin and maintains the cohesiveness of the silk cocoon, accounts for about 20–30% of the total mass of a cocoon [27,28]. Sericin is a hydrophilic protein composed of various amino acids such as glycine, alanine, and arginine. It contains a nitrogen content of approximately 17%, rendering it appropriate as a surface modification agent for electrode materials [28,29].

Herein, we propose a simple solid-state technique to prepare N-doped carbon wrapping on the NCA surface using sericin powder extracted by the degumming process. To optimize the suitable weight percentages of the N-doped carbon for effective surface wrapping, different weight percentages of sericin were used. The as-prepared N-doped carbon-coated NCA material was then physically and electrochemically characterized to identify the effect of N-doped carbon surface wrapping to overcome the disadvantages associated with NCA.

## 2. Materials and Methods

### 2.1. Sericin Synthesis

Sericin was extracted through the degumming of the silkworm cocoon. Initially, 0.02 M Na_2_CO_3_ was dissolved in 100 mL distilled water and allowed to boil on a hot plate at 100 °C. Then, the cocoon was added to it and boiled for 2–3 h to extract the sericin content from the cocoon [30,31]. After extraction of the sericin content, the pale yellowish solution was filtered to eliminate the remaining silk fiber after the sericin was dissolved. Then, the solution was dried in a convection oven at 80 °C for 12 h, and the sericin powder was obtained after drying.

### 2.2. Synthesis of Nitrogen-Doped Carbon Coated NCA (NC@NCA)

Using a simple solid-state method, NC@NCAs were synthesized. Approximately 2 g of commercial NCA (EcoPro BM, Ltd., Cheongju, Korea) was ground with sericin powder for approximately 1 h for thorough mixing. Subsequently, the mixed powder was heated at 500 °C for 3h in the presence of argon gas to get NC@NCA. For comparison, different amounts of sericin were used, such as 0.2, 0.3, and 0.4 weight percent, to prepare NC@NCA and designated as 0.2 NC@NCA, 0.3 NC@NCA, and 0.4 NC@NCA, respectively.

### 2.3. Physical Measurements

The crystal structure and phase of the as-synthesized samples were analyzed with an X-ray diffractometer (XRD, Bruker D8 Advance, Billerica, MA, USA, Cu-Kα radiation, λ = 1.5406 Å) in the 2θ range of 10°–70°. X-ray photoelectron spectroscopy (XPS, K-Alpha; Thermo Fisher, Waltham, MA, USA) was conducted to determine the constituent elements on the surface of the material. We also carried out high-resolution field-emission scanning electron microscopy (HR-FE-SEM, MERLIN-LEO SUPRA 55, Carl Zeiss, Jena, Germany) embedded with energy-dispersive X-ray spectroscopy (EDX, Ox-ford Instruments, Abingdon, UK) to determine the morphological characteristics and to conduct elemental analysis and elemental mapping.

### 2.4. Electrochemical Measurements

The obtained active materials were combined with Denka black as a conductive agent and polyvinylidene fluoride (PVDF) as a binder at a weight ratio of 80:10:10 to make the working electrode. Then, as a dispersion agent, an appropriate amount of N-methyl-2-pyrrolidone (NMP) was added to make a slurry. The prepared slurry was laminated on aluminum foil followed by room temperature drying for a day. The electrode was then dried in a convection oven at 120 °C for 5 h, lab-pressed, and punched into 14 mm diameter disks using a punching tool. Finally, the electrode was dried for 5 h at 100 °C in a vacuum oven. The as-produced electrode’s electrochemical performance was assessed in 2032-type coin cells, with lithium foil as the reference electrode, Celgard^®^ 2320 membrane as the separator, and 1 M LiPF_6_ in ethylene carbonate (EC) and diethyl carbonate (DEC) (1:1 vol. percent) as the electrolyte. The average mass loading of the cathode was 6 mg cm^−2^. The CR2032-type half-cells used to evaluate electrochemical performance were made in a glove box filled with Ar gas and aged for several hours. The electrochemical performance of the prepared cells was evaluated within a voltage range of 3.0–4.3 V using a cycler (ETH cycler, Hwaseong, Korea). Electrochemical impedance spectroscopy (EIS) was also conducted; the NCA cells completed for 200 cycles at a current density of 1 C were used and measured by applying an amplitude of 10 mV and a frequency range of 0.5 mHz–100 kHz. EIS measurements were carried out using an electrochemical workstation (Iviumstat, Ivium Technologies, Eindhoven, The Netherlands).

## 3. Results and Discussion

### 3.1. Physico-Chemical Characterizations

**Figure 1:**
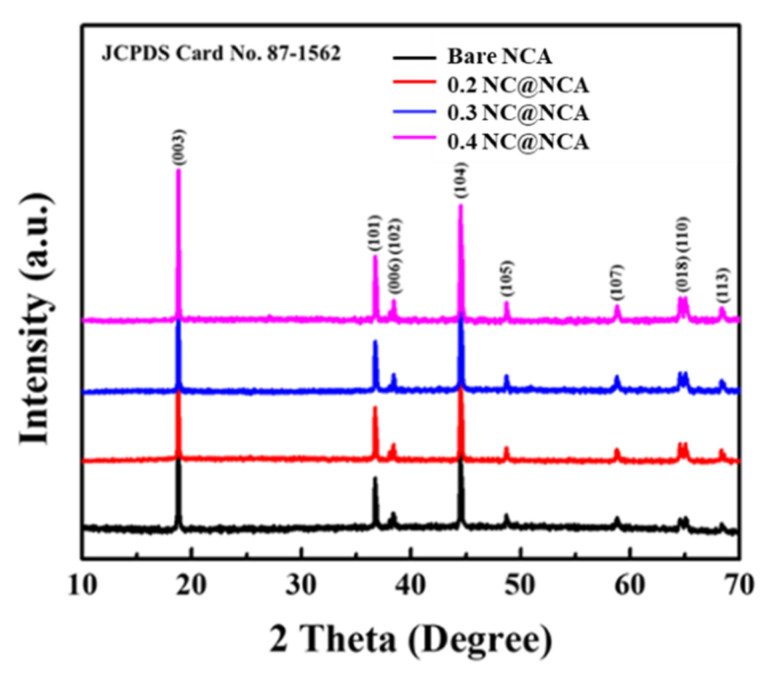
XRD patterns of bare NCA, 0.2 NC@NCA, 0.3 NC@NCA, and 0.4 NC@NCA.

The XRD patterns of bare NCA, 0.2 NC@NCA, 0.3 NC@NCA, and 0.4 NC@NCA are shown in Figure 1, and their equivalent lattice parameters are exhibited in Table 1; these diffraction patterns and lattice parameters are used to identify differences in crystal structure and crystallinity. In Figure 1, the XRD patterns of bare NCA, 0.2 NC@NCA, 0.3 NC@NCA, and 0.4 NC@NCA exhibited no differences, and all the peaks are well matched to JCPDS NO. 87-1562, which is indexed to α-NaFeO_2_ structure with R3m space group [32]. Furthermore, it is confirmed that the change of the lattice parameter did not appear significantly before and after coating, as indicated in Table 1. From these points, the N-doped carbon derived from sericin have no effect on the crystal structure of the bare NCA [33]. However, in terms of c/a values, there is little difference between the materials. The higher the ratio of c/a values, the higher the crystallinity and stability of NCA having a layered structure [34,35]. In this regard, it may be evident that 0.3 NC@NCA and 0.4 NC@NCA have higher crystallinity and stability than bare NCA. However, 0.2 NC@NCA has a lower c/a value than that of bare NCA, which means lower crystallinity and stability.

**Figure 2:**
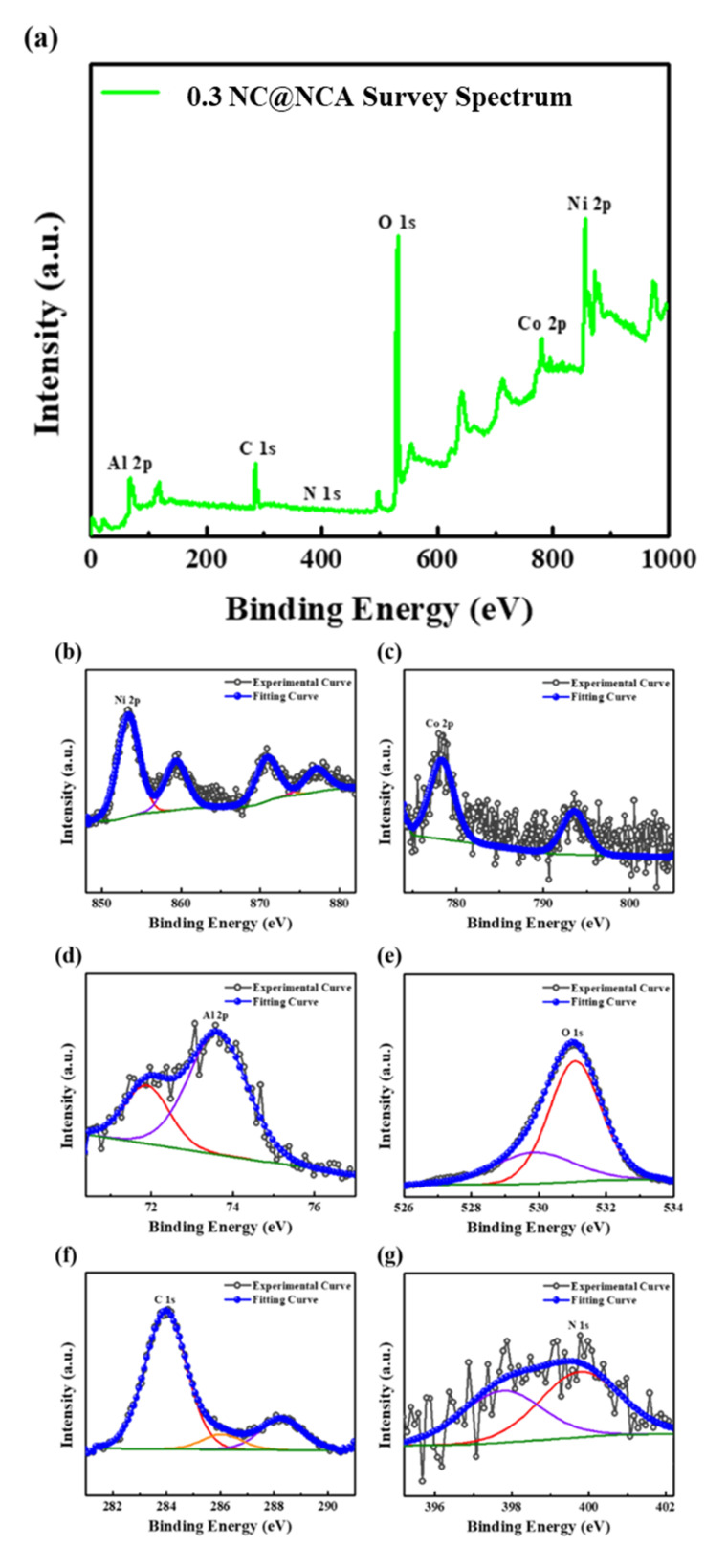
XPS spectra for 0.3 NC@NCA: (**a**) survey; (**b**) Ni 2p; (**c**) Co 2p; (**d**) Al 2p; (**e**) O 1s; (**f**) C 1s; and (**g**) N 1s.

XPS was conducted to study the chemical valence states and elemental composition of 0.3 NC@NCA. As shown in Figure 2a, the 0.3 NC@NCA XPS spectra exhibited the peaks of Ni, Co, Al, O, C, and N. The Ni 2p, Co 2p, Al 2p, and O 1s peaks fit well with the previous reports of the NCA [36,37,38]. Moreover, the C 1s and N 1s peaks are also observed, indicated by the N-doped carbon. Figure 2f displays the C 1s spectrum, which is fitted with peaks of C–C, C–O–C, and O–C=O bonds at binding energies of 284.0, 286.2, and 288.3 eV [39,40]. In Figure 2g, the N 1s spectra are fitted into two peaks for pyridinic N and pyrrolic N at binding energies of 397.8 and 399.8 eV [21,41]. The pyridinic N and pyrrolic N observed in 0.3 NC@NCA generate numerous active sites, which facilitate the rapid diffusion of lithium ions [42,43]. Consequently, the N-doped carbon layer contains pyridinic N and pyrrolic N, leading to improved electrochemical performance without disturbing the chemical states of NCA.

**Figure 3:**
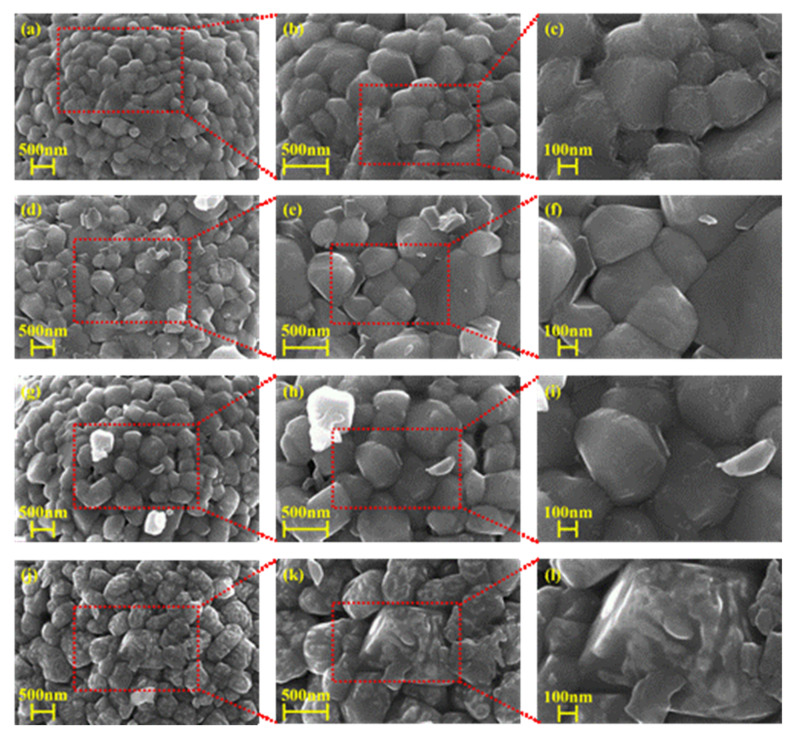
HR-FE-SEM images of (**a**–**c**) bare NCA; (**d**–**f**) 0.2 NC@NCA; (**g**–**i**) 0.3 NC@NCA; and (**j**–**l**) 0.4 NC@NCA.

Figure 3a–i show HR-FE-SEM images of bare NCA, 0.2 NC@NCA, 0.3 NC@NCA, and 0.4 NC@NCA. The surface of bare NCA, investigated by the HR-FE-SEM image, is observed as a rough surface. After the addition of sericin as the coating agent, the original shape of the materials is preserved for NC@NCA. Additionally, N-doped carbon on the surface of NC@NCA is clearly evident in the high-resolution images. In particular, in 0.3 NC@NCA, N-doped carbon particles are visible on the surface, and in 0.4 NC@NCA, more particles are noticeable on NCA. This indicates that sericin changes the surface of the NCA but does not affect the original spherical shape of the NCA.

**Figure 4:**
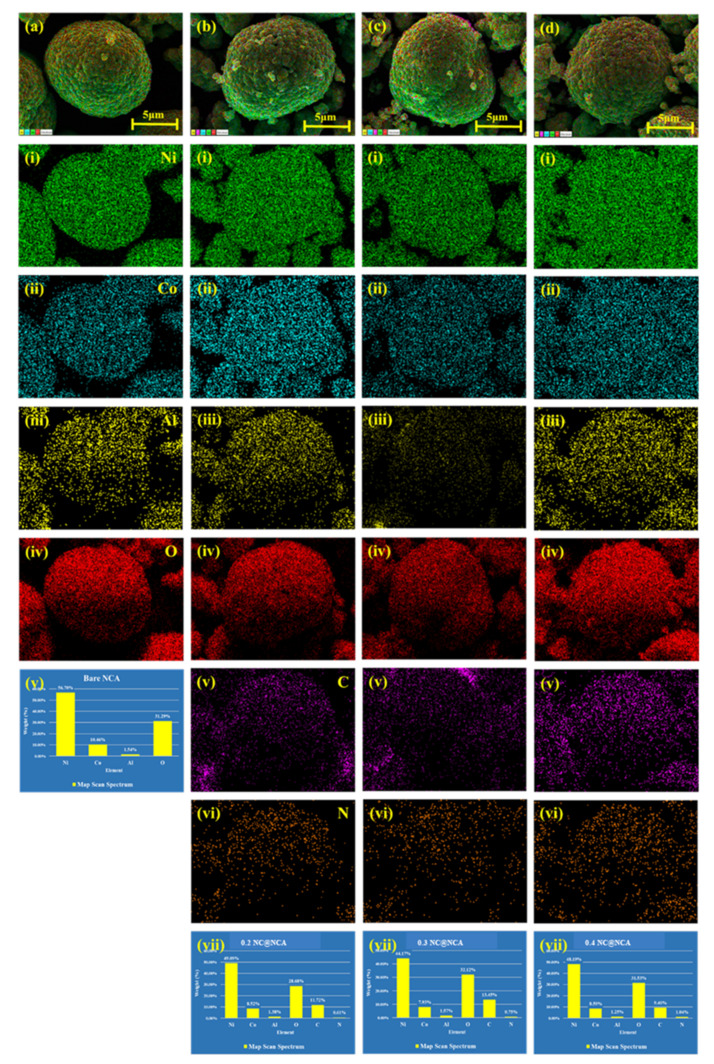
HR-FE-SEM images, elemental mapping spectra, and map scan spectra of (**a** (**i**–**v**)) bare NCA; (**b** (**i**–**vii**)) 0.2 NC@NCA; (**c** (**i**–**vii**)) 0.3 NC@NCA; and (**d** (**i**–**vii**)) 0.4 NC@NCA.

Figure 4a–d show the EDS elemental mapping and spectra mapping to further investigate the existence of various elements of bare NCA, 0.2 NC@NCA, 0.3 NC@NCA, and 0.4 NC@NCA. It is confirmed that Ni, Co, and Al are evenly and densely spread on the surface of them through each elemental mapping image. For 0.2 NC@NCA, 0.3 NC@NCA, and 0.4 NC@NCA, it is observed that nitrogen and carbon are also evenly distributed; an increase in the nitrogen content is noticeable as the weight percent of sericin increased, and the weight and atomic ratio of nitrogen for the materials are given in Table 2. The EDS mapping results confirmed that NCA is coated with N-doped carbon; as the weight percent of sericin increased, the thickness of the wrapping layer also grew.

### 3.2. Electrochemical Characterizations

**Figure 5:**
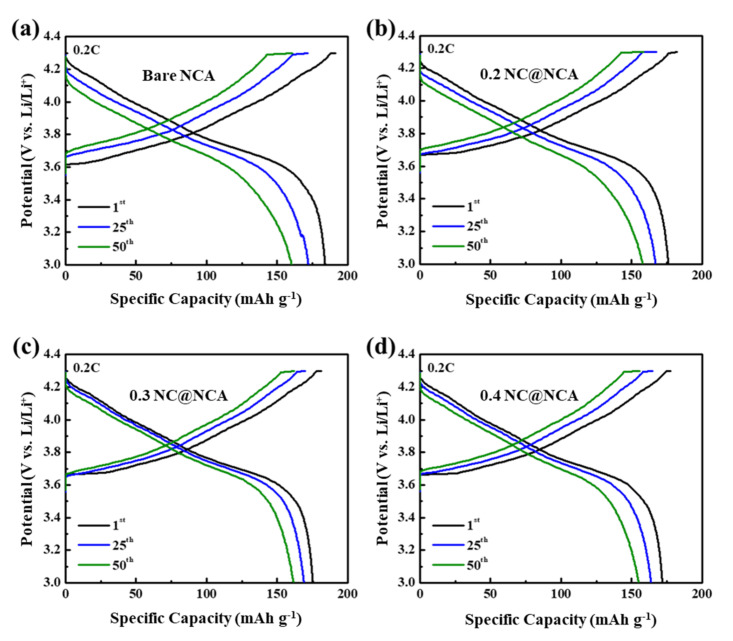
The galvanostatic charge/discharge potential profiles of (**a**) bare NCA; (**b**) 0.2 NC@NCA; (**c**) 0.3 NC@NCA; and (**d**) 0.4 NC@NCA.

The galvanostatic charge/discharge behavior analysis of bare NCA, 0.2 NC@NCA, 0.3 NC@NCA, and 0.4 NC@NCA was conducted using a cycler, as shown in Figure 5a–d. The evaluation process was conducted within a voltage range of 3.0–4.3 V with a current density of 0.2 C. Figure 5a exhibits that the bare NCA delivers the highest initial specific capacity among its counterparts. It is considered that the N-doped carbon wrapping would have impeded the ability of lithium ions to penetrate the NCA structure. For 50 cycles, it is verified that the decrease in specific capacity of the bare NCA is greater than its counterparts. Therefore, it is considered that the N-doped carbon wrapping minimizes side reactions with the electrolyte and polarization.

**Figure 6:**
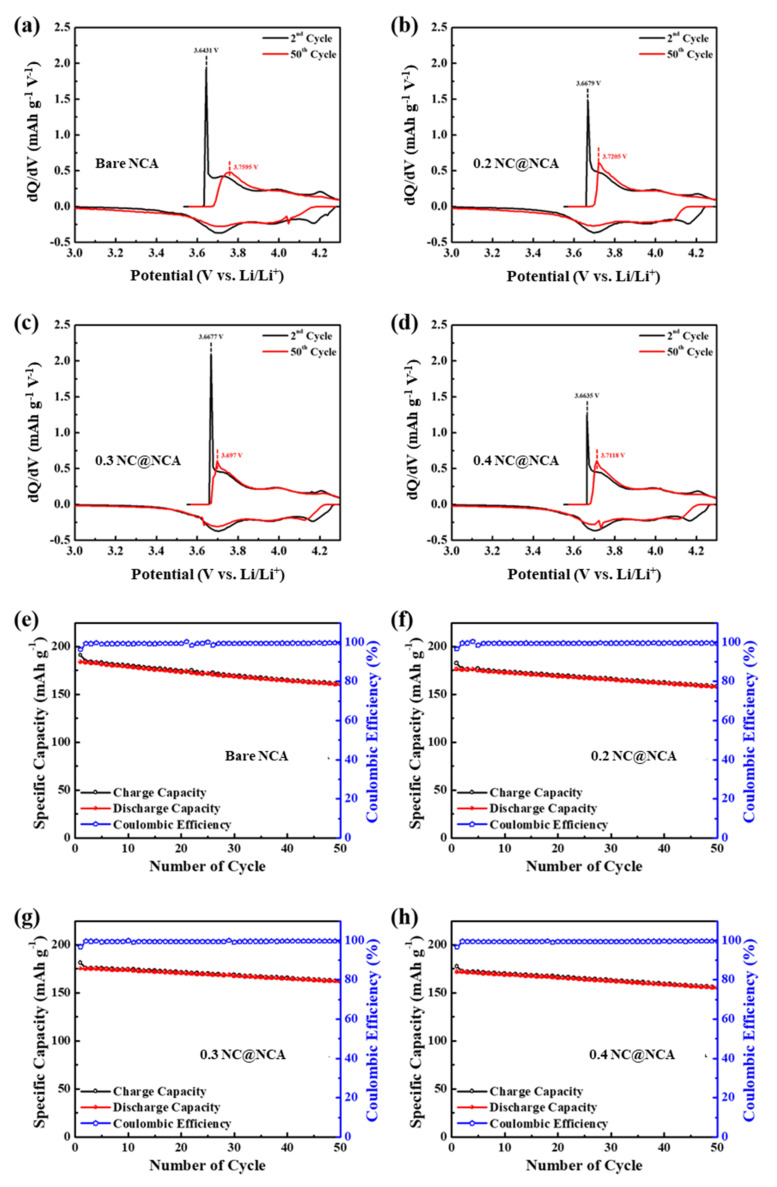
Differential capacity for (**a**) bare NCA, (**b**) 0.2 NC@NCA, (**c**) 0.3 NC@NCA, and (**d**) 0.4 NC@NCA; cycling performance profiles showing specific capacity and its coulombic efficiency at a current density of 0.2 C for (**e**) bare NCA, (**f**) 0.2 NC@NCA, (**g**) 0.3 NC@NCA, and (**h**) 0.4 NC@NCA.

Figure 6a–d show the differential capacity (dQ/dV) vs. voltage curve of the 2nd and 50th cycles at a current density of 0.2 C for bare NCA, 0.2 NC@NCA, 0.3 NC@NCA, and 0.4 NC@NCA. In the oxidation curve for the second cycle, the main sharp peak results from the formation of solid electrolyte interphase (SEI) on the NCA surface [44]. Furthermore, three pairs of clear peaks are visible, which are due to the multiple phase change from hexagonal (H1) to monoclinic (M), monoclinic (M) to hexagonal (H2) and hexagonal (H2) to hexagonal (H3) during cycling [45,46]. In the second cycle, the main oxidation peaks are 3.6431, 3.6679, 3.6677, and 3.6635 V, whereas, in the 50th cycle, the main oxidation peaks are shifted to 3.7595, 3.7205, 3.697, and 3.7118 V, respectively. For the bare NCA, the main oxidation peak shifted by 0.1164 V, and for the NC@NCA, the shifts in the main oxidation peaks are 0.0526, 0.0293, and 0.0483 V. Additionally, 0.2 NC@NCA, 0.3 NC@NCA, and 0.4 NC@NCA showed smaller shifts in oxidation peak compared with bare NCA; in particular, 0.3 NC@NCA experienced the smallest shift. The smaller shift in the oxidation peak means that the degree of reversibility during the cycling process improved [47]. That is, 0.2 NC@NCA, 0.3 NC@NCA, and 0.4 NC@NCA had better cycling reversibility than bare NCA; 0.3 NC@NCA, in particular, exhibited the best cycling reversibility.

The improved cycling reversibility was verified in Figure 6e–h; these figures exhibited the cycling performance of bare and N-doped carbon-coated NCA within a 3.0–4.3 V voltage range with a current density of 0.2 C. In the first cycle, bare NCA, 0.2 NC@NCA, 0.3 NC@NCA, and 0.4 NC@NCA exhibited specific discharge capacities of 183.95, 176.02, 175.31, and 171.78 mAh g^−1^, respectively. However, after the 50 cycles, the specific discharge capacity of bare NCA is 160.27 mAh g^−1^, whereas those of 0.2 NC@NCA, 0.3 NC@NCA, and 0.4 NC@NCA are 157.76, 161.63, and 155.10 mAh g^−1^, respectively. When investigated by capacity retention, bare NCA, 0.2 NC@NCA, 0.3 NC@NCA, and 0.4 NC@NCA are 87.13, 89.62, 92.20, and 90.29%, respectively; 0.2 NC@NCA, 0.3 NC@NCA, and 0.4 NC@NCA exhibited higher capacity retention compared with bare NCA. These outcomes imply that the N-doped carbon wrapping minimizes side reactions between the NCA and the electrolyte, improving the structural stability and cycling reversibility of NCA.

**Figure 7:**
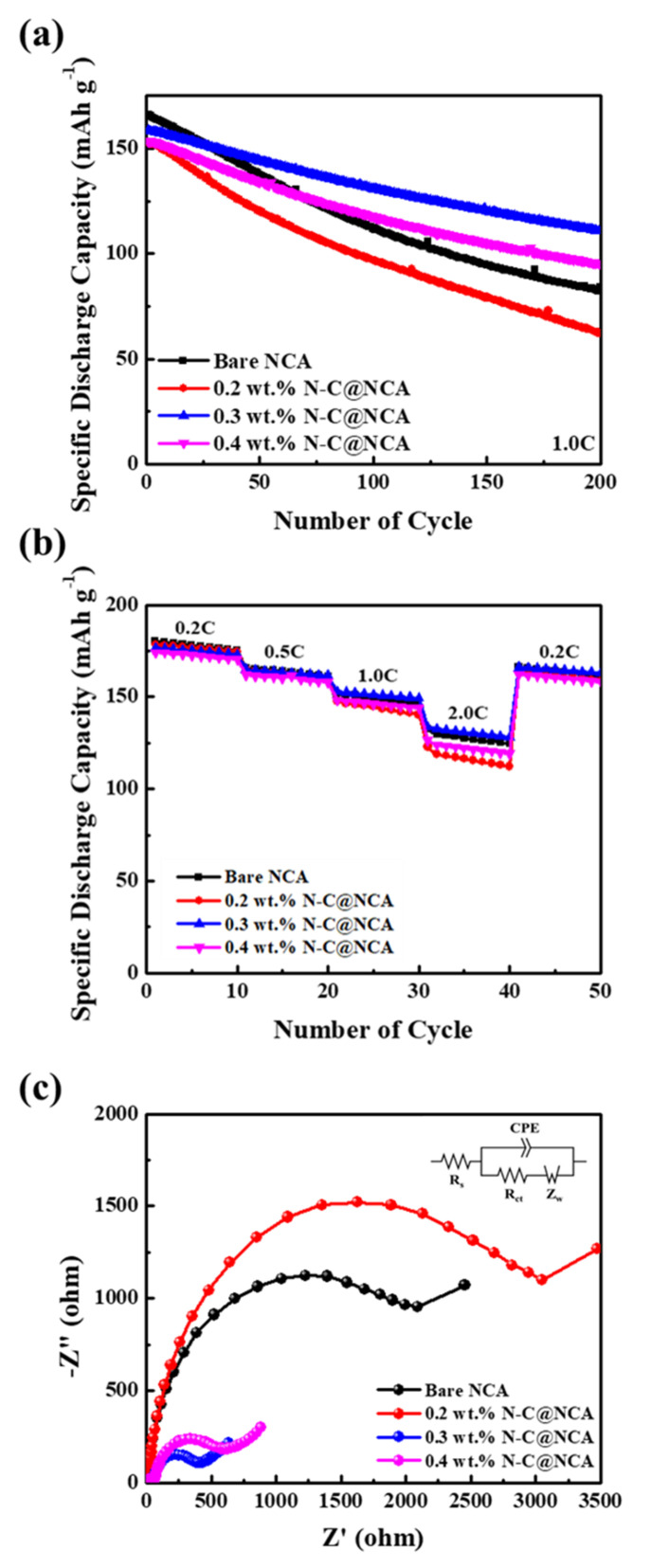
(**a**) Cycling performance at a current density of 1.0 C; (**b**) rate capability; (**c**) EIS analysis after 200 cycles at a current density of 1.0 C for bare NCA, 0.2 NC@NCA, 0.3 NC@NCA, and 0.4 NC@NCA.

Figure 7a shows the specific discharge capacity when 200 cycles of bare NCA, 0.2 NC@NCA, 0.3 NC@NCA, and 0.4 NC@NCA were conducted at a high current density of 1 C. In the initial cycle, the specific discharge capacity of bare NCA is higher than 0.2 NC@NCA, 0.3 NC@NCA, and 0.4 NC@NCA; however, after 200 cycles, the capacity retentions of 0.3 NC@NCA and 0.4 NC@NCA are 69.83% and 62.01%, respectively. This is higher than 0.2 NC@NCA with a capacity retention of 40.86% and a bare NCA of 50.65%. This low cycling stability in 0.2 wt.% N-C@NCA is attributed to the lower amount of sericin utilized. Therefore, it is considered that using a low amount of the coating agent could not form a stable coating layer to provide good cycling at a high current density, behaving instead as a minor variation. In addition, it is also considered that the low crystallinity and stability of 0.2 NC@NCA also contributed to the low-capacity retention at 1.0 C. For 0.4 NC@NCA, the capacity retention is higher than that of bare NCA; however, compared with 0.3 NC@NCA, the thicker coating layer impeded the movement of lithium ions and resulted in poor capacity retention.

Figure 7b shows the rate capability of bare NCA and 0.2 NC@NCA, 0.3 NC@NCA, and 0.4 NC@NCA. The process was conducted at various current densities from 0.2 C to 2.0 C. Bare NCA at a current density of 0.2 C exhibited the highest capacity, while at 0.5 C, it exhibited almost the same capacity as 0.3 NC@NCA. At current densities of 1.0 and 2.0 C, 0.4 NC@NCA exhibited a lower capacity than 0.3 NC@NCA. These results indicate that the 0.3 weight percent N-doped carbon wrapping is an effective coating layer capable of sufficiently protecting NCA during cycling at a high current rate.

To further analyze the effect of N-doped carbon on electrochemical performance, EIS was carried out for bare NCA, 0.2 NC@NCA, 0.3 NC@NCA, and 0.4 NC@NCA after 200 cycles at a 1 C current rate. Figure 7c shows that the semicircle in the high-frequency range, displayed in the Nyquist plot, is related to the charge transfer resistance (Rct), and the straight line in the low-frequency region is related to the Warburg resistance [48]. There is a large difference in charge transfer resistance, when comparing the bare NCA, 0.2 NC@NCA, 0.3 NC@NCA, and 0.4 NC@NCA. In numerical terms, the charge transfers resistances of the bare NCA, 0.2 NC@NCA, 0.3 NC@NCA, and 0.4 NC@NCA are 2091, 3049, 411, and 604 Ω, respectively. The charge transfer resistance is lowest for 0.3 NC@NCA, implying that this material showed the greatest improvement in ion and electron movement among them. This means that if 0.3 weight percent of sericin is used as the coating agent, the coating layer may serve as a protective layer for NCA and improve the movement of ions and electrons [49]. A table of comparison of previously reported similar studies with current work is presented in Table 3.

## 4. Conclusions

The N-doped carbon derived from sericin was successfully prepared using the solid-state method, and it was used to provide a coating layer for the NCA cathode material of LIBs. The influence of surface modification on the electrochemical characteristics of the NCA was characterized, and it was confirmed that this coating material is effective in improving electrochemical performance, particularly cycling stability. Specifically, 0.3 NC@NCA demonstrated the greatest improvements in cycling stability at current rates of 0.2 and 1.0 C as well as in rate capability. The measurement of charge transfer resistance using EIS confirmed that 0.3 NC@NCA showed the lowest charge transfer resistance. Based on these results, when 0.3 weight percent of sericin is used for the coating on NCA, it effectively suppresses side reactions with the electrolyte and surface degradation. Therefore, it could improve the structural stability of NCA, resulting in enhanced electrochemical performance, particularly in terms of cycling stability and rate capability.

## Figures and Tables

**Table 1:** Lattice parameters of bare NCA, 0.2 NC@NCA, 0.3 NC@NCA, and 0.4 NC@NCA.

	Bare NCA	0.2 NC@NCA	0.3 NC@NCA	0.4 NC@NCA
**a (Å)**	2.8636	2.8636	2.8625	2.8614
**c (Å)**	14.1532	14.1502	14.1643	14.1456
**c/a**	4.9425	4.9414	4.9482	4.9436

**Table 2:** Nitrogen weight ratio and nitrogen atomic ratio of bare NCA, 0.2 NC@NCA, 0.3 NC@NCA, and 0.4 NC@NCA.

	Bare NCA	0.2 NC@NCA	0.3 NC@NCA	0.4 NC@NCA
**Nitrogen Weight Ratio (wt.%)**	0	0.61	0.75	1.04
**Nitrogen Atomic Ratio (%)**	0	1.13	1.30	1.94

**Table 3:** Comparison of previously reported similar studies with current work.

Material	Carbon Source	Discharge Capacity (mAh g^−1^)	Capacity Retention (%)	Ref.
Carbon nanotube coating on NCA (CNT-NCA)	Carbon nanotubes	205 (at 0.1 C)	91 (at 0.1 C)	[50]
Carbon-coated LNCAO (LNCAO/C)	Sodium dodecyl sulfate	183 (at 0.1 C)	93 (at 0.1 C)	[51]
NCA-graphene	Graphene nanoplatelets	180 (at 0.5 C)	97 (at 0.5 C)	[52]
Nanoscale carbon coating on NCA (C@NCA)	Glucose	260 (at 1 C)	88 (at 1.0 C)	[53]
N-doped carbon-coated NCA (NC@NCA)	Sericin	161 (at 0.2 C)	92 (at 0.2 C)	*This work

## Data Availability

Not applicable.
